# Advanced Glycation End Products Impair Voltage-Gated K+ Channels-Mediated Coronary Vasodilation in Diabetic Rats

**DOI:** 10.1371/journal.pone.0142865

**Published:** 2015-11-12

**Authors:** Wen Su, Weiping Li, Hui Chen, Huirong Liu, Haixia Huang, Hongwei Li

**Affiliations:** 1 Department of Cardiology, Beijing Friendship Hospital, Capital Medical University, Beijing, PR China; 2 Department of Physiology and Pathophysiology, School of Basic Medical Sciences, Capital Medical University, Beijing, PR China; University of Colorado Denver School of Medicine, UNITED STATES

## Abstract

**Background:**

We have previously reported that high glucose impairs coronary vasodilation by reducing voltage-gated K^+^ (K_v_) channel activity. However, the underlying mechanisms remain unknown. Advanced glycation end products (AGEs) are potent factors that contribute to the development of diabetic vasculopathy. The aim of this study was to investigate the role of AGEs in high glucose-induced impairment of K_v_ channels-mediated coronary vasodilation.

**Methods:**

Patch-clamp recording and molecular biological techniques were used to assess the function and expression of K_v_ channels. Vasodilation of isolated rat small coronary arteries was measured using a pressurized myograph. Treatment of isolated coronary vascular smooth muscle cells (VSMCs) and streptozotocin-induced diabetic rats with aminoguanidine, the chemical inhibitor of AGEs formation, was performed to determine the contribution of AGEs.

**Results:**

Incubation of VSMCs with high glucose reduced K_v_ current density by 60.4 ± 4.8%, and decreased expression of K_v_1.2 and K_v_1.5 both at the gene and protein level, whereas inhibiting AGEs formation or blocking AGEs interacting with their receptors prevented high glucose-induced impairment of K_v_ channels. In addition, diabetic rats manifested reduced K_v_ channels-mediated coronary dilation (9.3 ± 1.4% *vs*. 36.9 ± 1.4%, *P* < 0.05), which was partly corrected by the treatment with aminoguanidine (24.4 ± 2.2% *vs*. 9.3 ± 1.4%, *P* < 0.05).

**Conclusions:**

Excessive formation of AGEs impairs K_v_ channels in VSMCs, then leading to attenuation of K_v_ channels-mediated coronary vasodilation.

## Background

Cardiovascular diseases are the primary causes of morbidity and mortality among patients with diabetes. It has been characterized that in conduit arteries, vascular dysfunction is largely due to the loss of modulatory role of the endothelium [1]. In contrast, vascular smooth muscle cells (VSMCs) have been reported to play a predominant role in the regulation of vascular tone for the microcirculation [2,3]. K^+^ channels in VSMCs take the principal responsibility for maintaining resting membrane potential and regulating smooth muscle tones [4]. We have previously demonstrated that voltage-gated K^+^ (K_v_) channels, especially the K_v_1 “Shaker-type” family, take responsibility for coronary vasodilation in rat small coronary arteries (RSCAs) [5,6]. K_v_ channels are involved in a number of physiological processes, including cAMP-dependent vasodilation [5,7]. Changes in the expression or activity of K_v_ channels often translate into a variety of vascular diseases including atherosclerosis [8], systemic and pulmonary hypertension [9,10], and especially diabetic vasculopathy [11]. In these diseases, K_v_ impairments associated with depolarizing shifts in VSMCs often result in a hypersensitivity to vasoconstrictor substances and increased level of vascular tone. Despite the importance of K_v_ channels in modulating vascular tone, mechanisms involved in impaired K_v_-mediated coronary microcirculation in diabetes remain poorly defined [5].

Advanced glycation end products (AGEs) are a group of cross-linked derivatives that are formed irreversibly in serum or tissues via non-enzymatic chemical reactions, due to hyperglycemia and oxidative stress [12]. There is accumulating evidence of the causal role for AGEs in the development of diabetic vasculopathy [13,14,15,16]. AGEs exert effects mainly by interacting with specific cell surface receptors, called receptor of advanced glycation products (RAGE) [17]. AGEs/RAGE axis increases inflammation and oxidative stress in many cell types including VSMCs, leading to vascular damage [18]. Retardation of AGEs formation with aminoguanidine (AG), the most extensively studied inhibitor of AGEs formation, has previously been shown to prevent diabetic vascular damage [19,20]. However, limited studies of the relationship between AGEs and altered K_v_ channel function have been conducted in the coronary VSMCs.

The aim of our study is to investigate whether AGEs would impair the activity and expression of K_v_ channels in VSMCs, and to further explore the role of AGEs in K_v_-mediated coronary dysfunction in diabetic animals.

## Methods

### Cell treatment

Primary rat coronary VSMCs were isolated according to published methods [21], and incubated in Dulbecco’s modified Eagle’s medium (DMEM, Gibco, USA) containing 10% fetal bovine serum (Gibco, USA), 100 U/mL penicillin, 100 mg/mL streptomycin, and 200 mmol/L L-glutamine for 48 h at 37^°^C. Cells were pretreated with AG (10 mmol/L), or anti-RAGE IgG (100 μg/mL), the RAGE neutralizing antibody, or vehicle for 30 min before incubation with 5.6 mmol/L (normal glucose) or 23 mmol/L (high glucose) D-glucose. To investigate the direct effect of AGEs, VSMCs were pretreated with anti-RAGE IgG (100 μg/mL) or vehicle for 30 min before stimulation with 100 ug/mL AGE-BSA for 48 h. The dose-dependent effect and osmotic influence of high glucose on coronary VSMCs have been previously evaluated [5,6,22,23], and the glucose concentration of 23 mmol/L was fixed for the following experiments. The concentrations of AGE-BSA and AG used were based on previous published studies [24,25].

### Animals

Six-week-old male Sprague-Dawley rats (Vital River, Beijing, China) weighing 180 to 200 grams were housed as described previously [23]. The rats were randomly divided into two parts in the beginning of the study. The controls were fed with regular chow (13 kcal% fat) for 4 weeks and injected with citrate buffer alone. Other rats received high-fat diet (58 kcal% fat with sucrose; Research Diets) for 4 weeks and then a single intraperitoneal injection of streptozocin (25 mg/kg, freshly prepared in 100 mmol/L citrate buffer, pH 4.5) after an overnight fast. Rats with blood glucose >16.7 mmol/L were considered to have diabetes [26,27]. Diabetic rats were treated with 1–3 U/day of insulin to prevent ketoacidosis. The rats were divided into four groups: control (*n* = 8), diabetes (DM, *n* = 8), control + AG (*n* = 8), or diabetes + AG (DM+AG, *n* = 8). The AG groups received 100 mg/kg/day AG dissolved in drinking water for 10 weeks. Animal protocols were based on the National Institutes of Health guidelines for care and use of laboratory animals, and approved by the Animal Care and Use Committee of Capital Medical University.

### Preparation of RSCAs and isometric force measurements

Rats were anesthetized with an intraperitoneal injection of sodium pentobarbital (60 mg/kg). RSCAs (internal diameter 150–200 μm) were dissected from the left ventricle and cut into 2 mm long rings. The endothelium was denuded with dry air. The efficacy of endothelial denudation was verified as previously reported [5]. The arterial rings were threaded on two stainless steel wires (40 μm in diameter) and mounted in 5 ml chambers of a multi wire myograph system (Model 610M, Danish Myo Technology, Aarhus, Denmark) filled with salt solution as previously described [5]. The physiological salt solution was continuously bubbled with 95% O_2_ and 5% CO_2_, and warmed to 37°C. Tension signals were attached to a PowerLab recording unit and saved to a Chart 7 for Windows software (AD Instruments Ltd, Aarhus, Denmark). After being mounted, the vessels were equilibrated for 1 h before normalization. The passive tension–internal circumference was determined by stretching to a transmural pressure of 60 mmHg as previously reported [6]. Vessels were precontracted with the thromboxane A_2_ analog U-46619 (10 nmol/L). Forskolin, an adenylyl cyclase activator, was used for eliciting cAMP-mediated vasodilation [5,28]. Dilations to forskolin (10^−10^–10^−6^ mol/L) were compared in arterial rings before and after the application of 4-aminopyridine (4-AP), a selective blocker of K_v_ channels. 4-AP (3 mmol/L) was added to the chambers and incubated for 20 min before dose-response curves were recorded.

### Patch-Clamp recording of K^+^ Currents

Standard pulse protocols were used to assess the whole-cell and voltage-gated K^+^ currents as detailed previously [5]. Briefly, K^+^ currents were generated by a series of 400 ms depolarizing pulses from -60 mV to +60 mV in 10 mV increments. K_Ca_ currents were minimized by using recording solutions with a low Ca^2+^ concentration (10 nmol/L) and adding 100 nmol/L iberiotoxin in the bath solution. Seal resistance was 2 to 10 GΩ. K_v_ current was defined by subtracting outward currents recorded in the presence of 3 mmol/L 4-AP from outward currents recorded in drug-free bath solution. Current density of K_v_ channels was calculated to normalize for cellular membrane area. Hyperpolarizing steps of 10 mV were averaged to measure cell capacitances and leak compensation values. Membrane currents were recorded with an EPC-9 amplifier and Pulse software (Heka Elektronik, Germany). Pulses were generated by a digital-to-analog converter controlled by Pulse software.

### Detection of AGEs

Serum samples and culture media were collected and AGEs levels were analyzed using an AGEs ELISA kit (MyBioSource, USA) according to the manufacturer's instruction.

### Western blotting

Western blotting in VSMCs and coronary tissues was performed as previously reported [29]. The following antibodies were used: anti-K_v_1.2, anti-K_v_1.5, anti-RAGE, and anti-β-actin (all from Abcam, U.K.).

### Quantitative real-time (RT)-PCR

RNA isolation and cDNA generation were done as described previously [23]. Gene expression of K_v_1.2 and K_v_1.5 was assessed by quantitative RT-PCR. Primers used were as follows: K_v_1.2 sense 5′-CATTTTGTACTACTACCAGTC-3′, antisense 5′-GGAGTGTCTGGACAACTTGA-3′; K_v_1.5 sense 5′-TGAGCAGGAGGGGAATCAGA-3′, antisense 5′-ACACCCTTACCAAGCGGATG-3′; β-actin sense 5′-CCCATCTATGAGGGTTACGC-3′, antisense 5′-TTTAATGTCACGCACGATTTC-3′. ΔΔCT values were calculated as previously described [23]. Gene expression was normalized with β-actin and reported as ratios compared with the level of expression in untreated control group, which was given an arbitrary value of 1.

### Chemicals

AGE-BSA for VSMCs incubation was purchased from Merck Millipore. Endotoxin levels were found to be less than 0.8 EU/mg protein with the Limulus amebocyte assay (E-Toxate kit, Sigma, USA). Anti-RAGE IgG was from R&D system (Minneapolis, USA). All the other chemicals were purchased from Sigma (St Louis, USA).

### Statistical analysis

The presented values were expressed as mean ± SD. Comparisons among different groups were performed by one-way ANOVA followed by Bonferroni test. The significance was regarded as *P* < 0.05.

## Results

### AGEs are required for high glucose-induced K_v_ channel dysfunction in VSMCs

**Fig 1:**
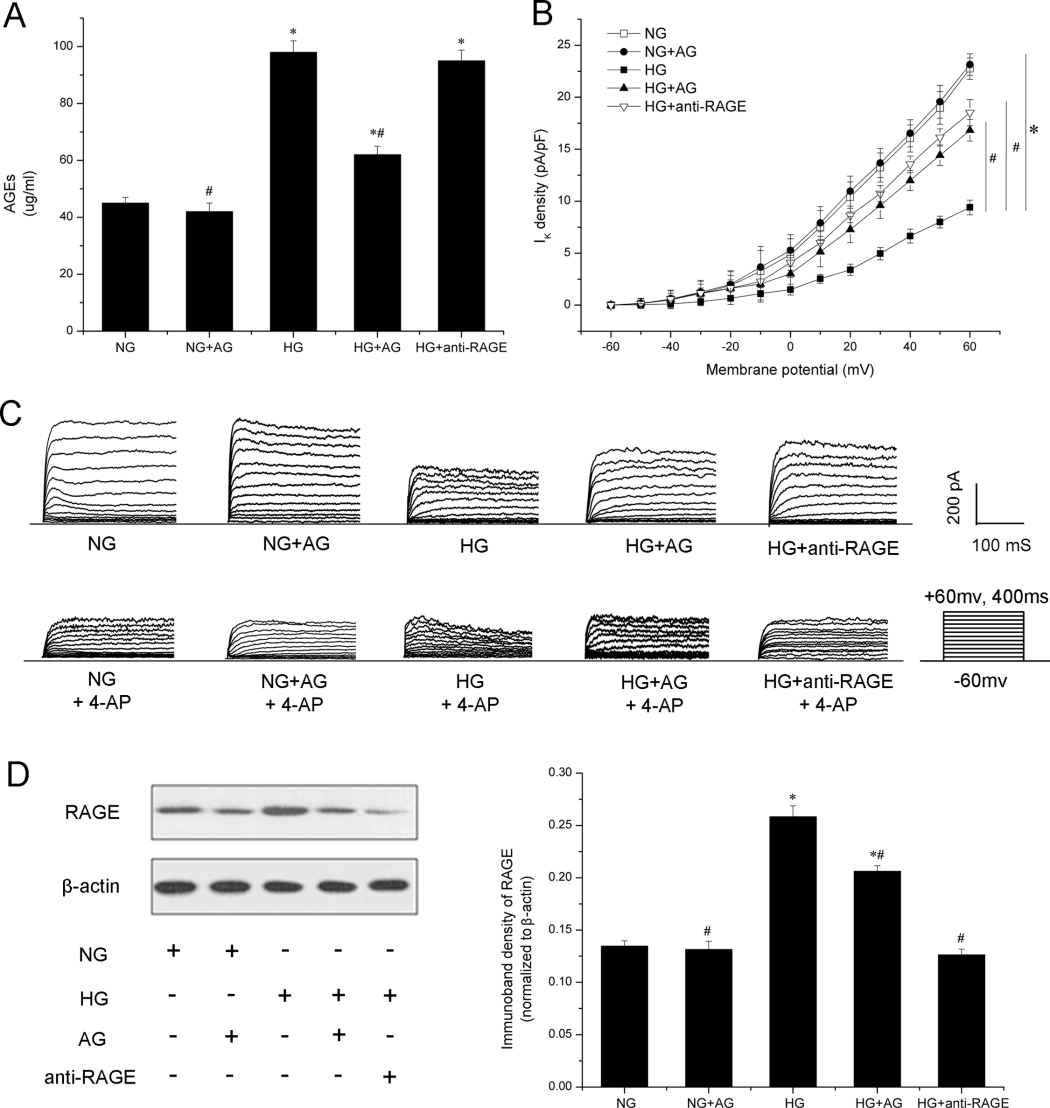
The role of AGEs in high glucose-induced voltage-gated K^+^ (K_v_) channels dysfunction in vascular smooth muscle cells (VSMCs). A: Overproduction of AGEs in high glucose was blunted by treatment with aminoguanidine (AG). B: I-V relationships of K_v_ current density in VSMCs. *n* = 6 for independent cells in each group. C: Sample traces of whole cell K^+^ currents recorded before and after incubation with 3 mmol/L 4-aminopyridine (4-AP). K^+^ currents were generated by 10-mV incremental depolarizing steps from -60 to +60 mV. D: Expression of receptor of advanced glycation products (RAGE) was determined by western blotting. Pretreatment with anti-RAGE decreased available RAGE for AGEs to bind. * *P* < 0.05 *vs*. normal glucose (NG). # *P* < 0.05 *vs*. high glucose (HG).

VSMCs were incubated with AG, followed by high glucose treatment for 48 h. As Fig 1A shows, AGEs were increased to approximately twofold by treatment with high glucose. In contrast, AG blunted the production of AGEs. Fig 1B and 1C show sample traces of whole cell K^+^ currents generated by 10-mV incremental depolarizing steps from -60 to +60 mV in VSMCs from RSCAs. Current-voltage relations averaged from six cells verified that high glucose reduced K_v_ current density by 60.4 ± 4.8%, whereas AG prevented the high glucose-induced impairment on K_v_ activity by 56.9 ± 7.8% (Fig 1B and 1C). Treatment with AG had no obvious effect on K_v_ activity in normal glucose-incubated cells.

Given the role of RAGE in AGEs-induced intracellular signaling cascades, the role of RAGE in K_v_ dysfunction was also investigated. VSMCs were pretreated with anti-RAGE IgG, before treatment with high glucose. Expression of RAGE was increased by high glucose treatment as compared with normal glucose group, whereas anti-RAGE IgG prominently decreased available RAGE for AGEs to bind (Fig 1D). Patch clamp results showed that anti-RAGE reversed the high glucose-induced suppression of K_v_ current density by 69.3 ± 9.5% (Fig 1B and 1C). These data suggest that high glucose-reduced K_v_ current density in VSMCs is mainly mediated via AGEs.

### AGEs mediate high glucose-induced downregulation of K_v_ channel expression

**Fig 2:**
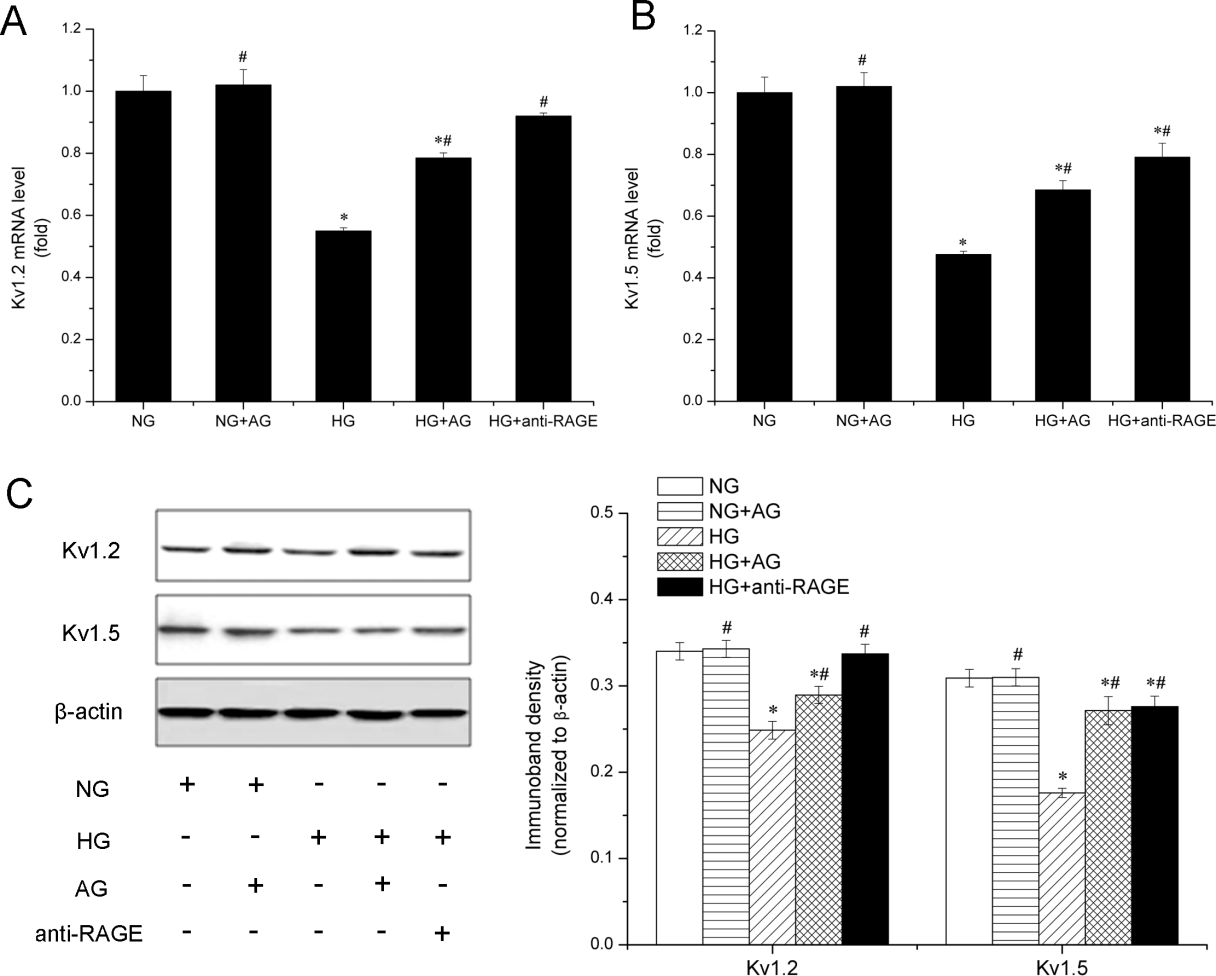
The role of AGEs in high glucose-induced downregulation of voltage-gated K^+^ (K_v_) channels expression in vascular smooth muscle cells (VSMCs). VSMCs were incubated with Aminoguanidine (AG) or anti-receptor of advanced glycation products (RAGE) for 30 min, followed by high glucose treatment, and expression of K_v_1.2 and K_v_1.5 at the gene and protein level was evaluated by quantitative real-time-PCR (A and B) and western blot analysis (C). * *P* < 0.05 *vs*. normal glucose (NG). # *P* < 0.05 *vs*. high glucose (HG).

The change of high glucose-induced K_v_ channel expression was further investigated. VSMCs were incubated with AG, followed by high glucose treatment, and the expression of K_v_1.2 and K_v_1.5 was evaluated by RT-PCR and western blot analysis. As Fig 2 shows, high glucose significantly decreased expression of K_v_1.2 and K_v_1.5 at the gene and protein level, as compared with normal glucose group. Treatment with AG prevented high glucose-induced impairment of K_v_1.2 expression. Additionally, anti-RAGE reversed the change of K_v_1.2 observed in high glucose, approaching the level observed in control. The expression of K_v_1.5 was also increased in AG and anti-RAGE groups at the gene and protein level, as compared with high glucose group, suggesting that AGEs are required for high glucose-induced impairment of K_v_1.2 and K_v_1.5 expression.

### AGEs impair K_v_ current and expression via interacting with RAGE

**Fig 3:**
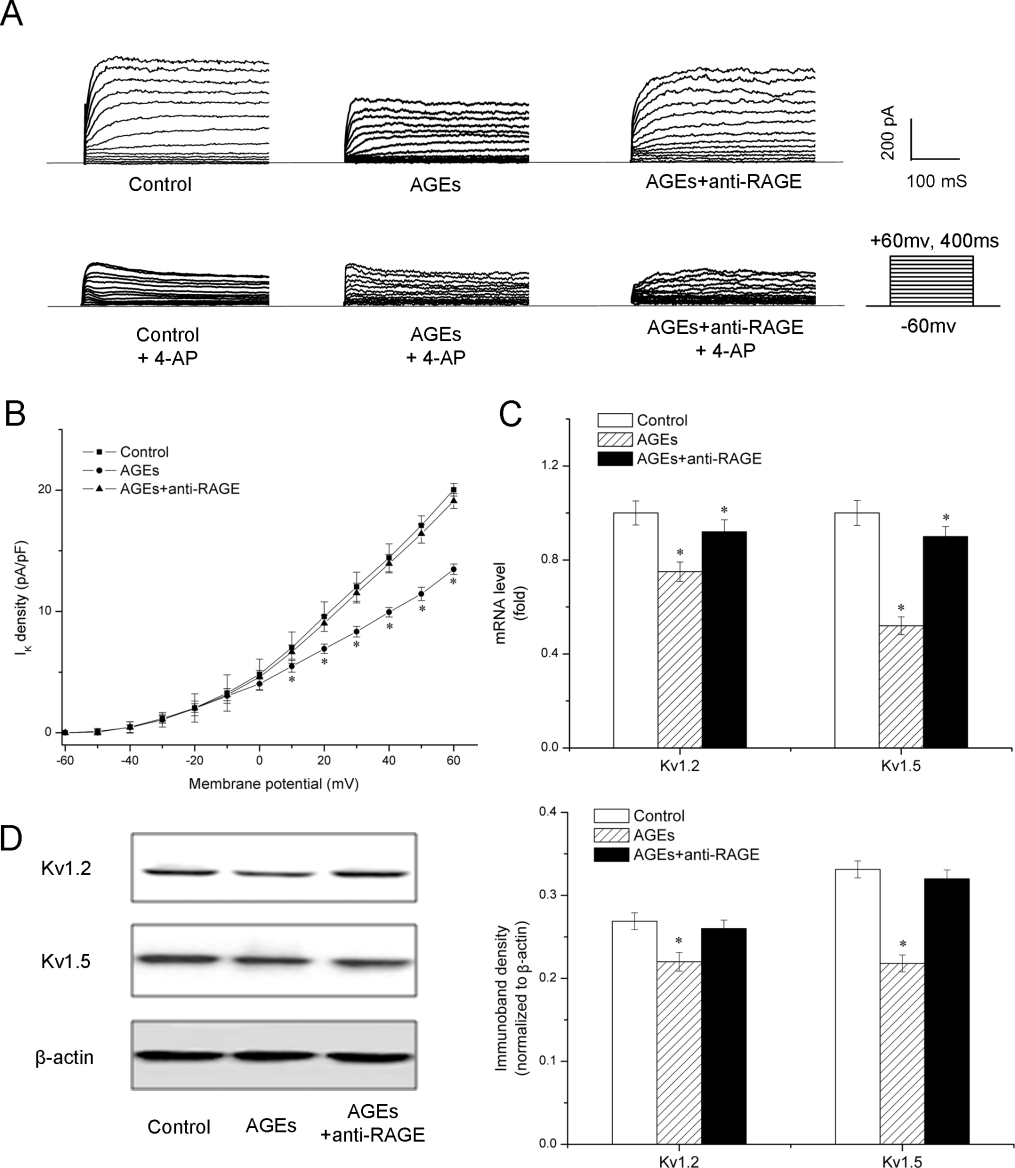
AGEs impair voltage-gated K^+^ (K_v_) current and expression via interacting with receptor of advanced glycation products (RAGE). A: Sample traces of whole cell K^+^ currents recorded before and after incubation with 3 mmol/L 4-aminopyridine (4-AP). K^+^ currents were generated by 10-mV incremental depolarizing steps from -60 to +60 mV. B: I-V relationships of K_v_ current density in vascular smooth muscle cells. *n* = 6 for independent cells in each group. C and D: After treatment with AGEs alone or AGEs plus anti-RAGE, expression of K_v_1.2 and K_v_1.5 at the gene and protein level was evaluated by quantitative real-time-PCR (C) and western blot analysis (D). * *P* < 0.05 *vs*. Control.

To further establish a role for AGEs in high glucose-induced K_v_ impairment, a direct effect of AGEs on K_v_ channels was investigated. VSMCs were pretreated with anti-RAGE or vehicle before treatment with AGE-BSA. AGE-BSA was incubated with VSMCs for 48 h followed by patch clamp, RT-PCR and western blot analysis. Treatment with AGEs was associated with a 34% reduction of K_v_ current density compared with control (Fig 3A and 3B). K_v_1.5 channel expression at the gene and protein level was significantly decreased (Fig 3C and 3D). K_v_1.2 also appeared reduced after AGEs treatment, as compared with control, albeit with a less dramatic change than K_v_1.5. Pretreatment with anti-RAGE blocked these modifications caused by AGEs, indicating that AGEs, independent of glucose concentrations, can impair K_v_ current and expression via interacting with RAGE.

### K_v_ channels-mediated coronary dilation is impaired in diabetic rats

**Fig 4:**
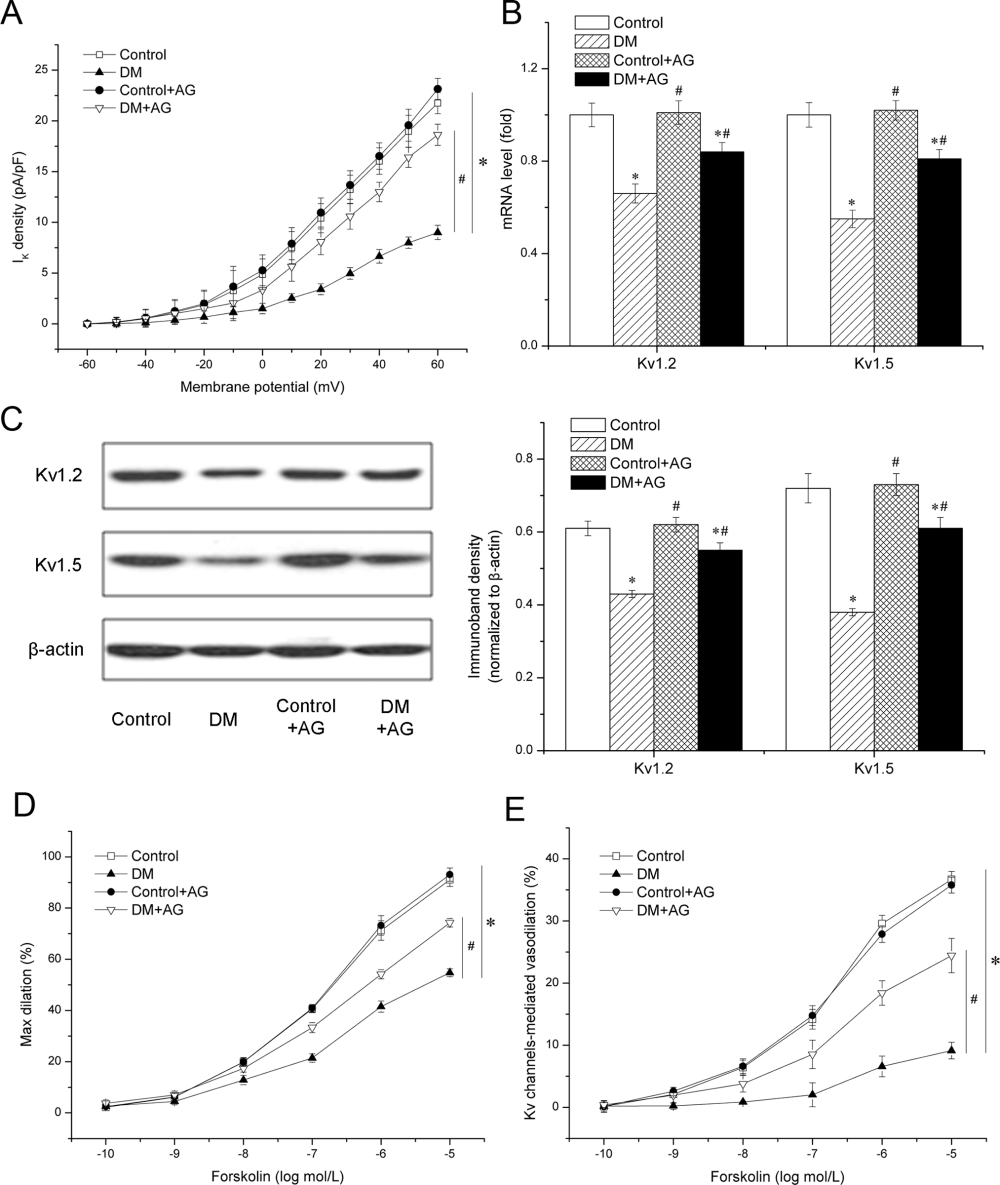
Role of AGEs in impaired K_v_ channels-mediated coronary vasodilation in diabetic rats. Control and Diabetic rats were treated with aminoguanidine (AG) or vehicle for 10 weeks. Rat small coronary arteries (RSCAs) were isolated from different rat groups. A: K_v_ current density in vascular smooth muscle cells isolated from different rat groups. *n* = 6 for independent cells in each group. B and C: Expression of K_v_1.2 and K_v_1.5 at the gene and protein level was evaluated by quantitative real-time-PCR (B) and western blot analysis (C). D: Dose-dependent dilation to forskolin in RSCAs was measured using a pressurized myograph. E: After incubation of RSCAs with 3 mmol/L K_v_ inhibitor 4-aminopyridine (4-AP) for 20 min, dilations to forskolin in all rat groups were significantly reduced. K_v_ channels-mediated vasodilation was defined as the difference between dilations measured before and after incubation with 4-AP. *n* = 8 for independent arterial rings in each group. * *P* < 0.05 *vs*. Control. # *P* < 0.05 *vs*. diabetic group (DM).

Diabetes-induced impairment of K_v_-mediated vasodilation in RSCAs was determined. Diabetic rats were confirmed and coronary arteries were isolated. Patch-clamp recording and molecular biological techniques revealed reduced K_v_ current density and decreased expression of both K_v_1.2 and K_v_1.5 in diabetic rats (*P* < 0.05 *vs*. control, Fig 4A, 4B and 4C), which was consistent with the results *in vitro*. RSCAs were mounted in an arteriograph. To exclude the potential role for endothelial involvement, coronary dilation was compared in the absence of endothelium. Fig 4D illustrates that forskolin elicited vasodilation of RSCAs in a dose-dependent manner. Maximal dilations to forskolin in RSCAs of diabetic rats were reduced (54.6 ± 1.6 *vs*. 91.1 ± 2.1%, *n =* 8 for independent arterial rings in each group, *P* < 0.05 *vs*. control). These results suggest that diabetes impairs vasodilation mediated by cAMP. Impaired cAMP-mediated dilation in DM can be explained by vascular changes independent of the endothelium.

The requirement for K_v_ in diabetes-induced vascular dysfunction was further investigated. Maximal dilations to forskolin were reduced by 3 mmol/L 4-AP, suggesting that the cAMP-mediated dilation in these vessels is predominantly regulated by K_v_ channels. 4-AP blocked vasodilation in both control and DM group. Moreover, the 4-AP-sensitive component of forskolin-induced dilation in DM group was reduced (9.3 ± 1.4% *vs*. 36.9 ± 1.4%, *P* < 0.05 *vs*. control, Fig 4E), suggesting that K_v_ channels-mediated coronary dilation is impaired in diabetic rats.

### Diabetes-induced impairment of vasodilation requires AGEs formation in rat coronary microvessels

To evaluate the effect of AGEs *in vivo*, diabetic rats were randomized to be treated with AG or vehicle. After 10 weeks, coronary arteries were harvested. As Table 1 shows, treatment with AG had no effect on weight, blood pressure, and glucose level. However, AG decreased serum AGEs. Both function and expression of K_v_ were increased by AG treatment, compared with the DM group (Fig 4A, 4B and 4C). In non-diabetic controls, although AG treatment lowered AGEs level, it failed to increase K_v_ expression.

**Table 1:** Clinical characteristics of control and diabetic rats.

	Control	DM	Control+AG	DM+AG
Weight, g	424.1 ± 1.6	395.1 ± 1.8*	425.5 ± 1.6^#^	395.3 ± 3.2*
Systolic BP, mmHg	124.0 ± 1.3	133.3 ± 2.6*	125.0 ± 1.9^#^	131.3 ± 2.5*
Diastolic BP, mmHg	84.5 ± 1.9	88.5 ± 1.9*	84.6 ± 1.9^#^	88.0 ± 1.6*
Glucose, mmol/L	5.8 ± 0.3	23.4 ± 1.6*	5.9 ± 0.2^#^	23.3 ± 1.9*
AGEs, ug/mL	49.8 ± 2.9	99.1 ± 2.7*	42.8 ± 2.4^#^	61.4 ± 1.9* ^#^

Values are presented as mean ± SD. *n* = 8 for each group. BP, blood pressure. AGEs, advanced glycation end products. AG, aminoguanidine.

* *P* < 0.05 *vs*. control.

^#^ *P* < 0.05 *vs*. diabetic group (DM).

Forskolin-elicited relaxation was significantly altered in RSCAs from AG-treated diabetic rats compared to vehicle-treated diabetic rats, as assessed with a myograph. Maximal dilations to forskolin in RSCAs of AG-treated diabetic rats were improved (74.3 ± 1.6 *vs*. 54.6 ± 1.6%, *n =* 8 for independent arterial rings in each group, *P* < 0.05 *vs*. DM, Fig 4D). In addition, AG treatment partially reversed the decrease of K_v_-mediated coronary vasodilation (24.4 ± 2.2 *vs*. 9.3 ± 1.4%, *n =* 8 for independent arterial rings in each group, *P* < 0.05 *vs*. DM, Fig 4E). The data shown above suggest that AGEs are upstream regulators for K_v_ impairment, which mediates diabetes-induced reduction in coronary dilation.

## Discussion

The aim of this study was to determine the mechanism of coronary dysfunction in response to diabetes, and in particular, the role of AGEs. High glucose reduced approximately half of the K_v_ current density in VSMCs, whereas the reduction was almost prevented by AG, the chemical inhibitor of AGEs formation. Furthermore, AG prevented high glucose-induced impairment of K_v_ expression. Finally *in vivo* data from diabetic rat coronary microvessels further support a role for AGEs in K_v_-mediated coronary dysfunction. These studies strongly suggest that at least part of the coronary dysfunction in diabetes is mediated via impairment of K_v_ channels and excessive formation of AGEs takes major responsibility in this process.

The present study demonstrate that AGEs impaired cAMP-mediated relaxation in rat coronary microvessels and the mechanism involved impairment of K_v_ channels in VSMCs. K_v_ channels have been previously shown to be impaired in various pathologic conditions, such as hypertension [10], and hypercholesterolemia [30]. Some of the effects observed in DM rats can be due to other factors, such as the raised blood pressure. However, exposure of VSMCs to AGE-BSA *in vitro* could directly evaluate the effect of AGEs on K_v_ channels without confounding influences *in vivo*. Patch clamp studies revealed that AGEs reduced a 4-AP sensitive component of K^+^ current, suggesting that K_v_ channels are susceptible to AGEs inhibition. In addition, our data showed that AGEs downregulated K_v_1.2 and K_v_1.5 expression at the gene and protein level. Our previous study presented that short-term exposure of RSCAs to high glucose for 24 h enhanced nitration of K_v_ channels without changing the expression of K_v_1.2 and K_v_1.5 [6]. The difference between these two results may be ascribed to the different methods and exposure time. Incubation of RSCAs *in vitro* may not produce the same degree of stimulation to K_v_ channels as in direct incubation of VSMCs. Our results are consistent with a model in which AGEs downregulated the expression of K_Ca_2.3 and K_Ca_3.1 channels in human umbilical vein endothelial cells and damaged K_Ca_2.3 and K_Ca_3.1-mediated relaxation in small mesenteric arteries [31]. Because K_v_ channels are critical in vascular function [8,9], they represent potential therapeutic targets to restore normal levels of vascular reactivity.

Overproduction of AGEs has been reported to contribute to endothelial dysfunction in type 2 diabetes. A previous study by Gao and coworkers showed that impaired coronary vasodilator response to acetylcholine was restored by blockade of AGEs/RAGE [13]. AGEs-induced inactivation of nitric oxide and impairment of endothelium-dependent vasodilation were also seen in rat aortas [1] and mesenteric resistance arteries [14]. Here, we further examined the mechanism of AGEs-mediated vascular dysfunction, focusing on the potential role of endothelium-independent vasodilation. The mechanical force leading to vasoconstriction is exerted by smooth muscle cells, which were reported to play a predominant role in the regulation of pressure-induced vasodilation [32]. We previously compared coronary dilation to forskolin in the presence and absence of endothelium. Endothelial denudation did not reduce dilation to forskolin. Furthermore, the reduction in forskolin-induced dilation in high glucose was similar in intact and denuded vessels [5]. The results presented herein further strengthen this link and suggest that overproduction of AGEs in high glucose environment leads to the impairment of K_v_ channels, regulating endothelium-independent vasodilation.

The AGEs/RAGE axis appears to play a major role in vascular dysfunction in diabetes [33,34]. In our study, RAGE expression was increased by high glucose treatment, which was consistent with previous studies showing that RAGE expression can be triggered by multiple molecular ligands including AGEs concomitantly present in high glucose incubation [22,35]. Since AGEs formation is a non-enzymatic reaction, both K_v_1.2 and K_v_1.5 under high glucose concentration may be glycated to form AGEs, and their glycation will surely impair the K_v_ channels. However, AGEs have been reported to do their job both through direct and indirect ways [12]. In the former, AGEs exert bad effects via cross linking of important proteins. On the other hand, AGEs bind to RAGE, thereby inducing oxidative stress [18,36,37,38,39], leading to K_v_ channel impairment and vascular damage [6,40,41,42]. Therefore, we used anti-RAGE to block AGEs binding with RAGE. As Fig 1 and Fig 2 show, pretreatment with anti-RAGE almost inhibited high glucose-induced impairment of K_v_ channels without changing the level of AGEs. So, we conclude that the impairment of K_v_1.2 and 1.5 happens downstream of the binding of AGEs and RAGE. It seems unlike that K_v_1.2 and K_v_1.5 proteins are itself glycated by high glucose. This finding highlights that AGEs-induced K_v_ impairment is primarily mediated through RAGE activation. Thus, inhibiting AGEs formation or blocking AGEs interacting with RAGE may be a potential therapeutic target for K_v_-mediated coronary dysfunction in diabetic patients.

The interaction of AGEs/RAGE can activate a diverse array of intracellular signaling pathways including p21^ras^, MAPK, and NF-κB that stimulate oxidative stress [18,36,37,38,39]. Increased oxidative stress has been described to downregulate K_v_ channels both at the protein and mRNA level [40,41,42]. In addition, AGEs/RAGE interaction has been shown to downregulate PPARγ, a member of the nuclear hormone receptor superfamily, which was proposed to be a regulator of transcriptional regulation of K_v_ channel expression in retinal arterioles [43,44]. Expression and function of K_v_ channels vary between different vascular tissues [45]. We previously reported that peroxynitrite, formed by the interaction of superoxide and nitric oxide, impaired K_v_ channel function by nitrating the K_v_ channel protein in coronary arterioles [6]. Therefore, we speculate that AGEs/RAGE-augmented oxidative stress may play a role in impaired K_v_ channels in coronary vascular smooth muscle cells. However, further studies should be done to advance the understanding of mechanisms involved in AGEs/RAGE-downregulated K_v_ channel expression.

Several limitations of this study should be acknowledged. First, other families of K_v_ channels have also been shown to be expressed in VSMCs and might also be affected in this diabetic model. We here only focus on the representative channel types that were reported to be involved in vasodilation in our previous studies [5,6]. Future studies will examine the changes of other VSMC K_v_ channels in diabetes-induced vascular dysfunction. Second, the relevance of our findings is limited in the specificity of the inhibitors. Although AG is the most extensively studied inhibitor of AGEs formation, and rigorous control experiments were performed, genetic approaches will still be required to confirm the effect of AGEs/RAGE axis in K_v_ impairment.

## Conclusions

AGEs impair K_v_ channels-mediated coronary vasodilation in diabetic rats. Our study demonstrates for the first time that high glucose-stimulated excessive AGEs impair K_v_ channels in VSMCs, and supports a role for K_v_ channels in the regulation of vascular function in diabetic rats. These results may provide a novel insight into the mechanisms of diabetic coronary dysfunction, and have clinical implications for the treatment of vascular complications in diabetic patients.
